# Development of iRGD-Modified Peptide Carriers for Suicide Gene Therapy of Uterine Leiomyoma

**DOI:** 10.3390/pharmaceutics13020202

**Published:** 2021-02-02

**Authors:** Anna Egorova, Sofia Shtykalova, Alexander Selutin, Natalia Shved, Marianna Maretina, Sergei Selkov, Vladislav Baranov, Anton Kiselev

**Affiliations:** 1 Department of Genomic Medicine, D.O. Ott Research Institute of Obstetrics, Gynecology and Reproductology, Mendeleevskaya Line 3, 199034 Saint-Petersburg, Russia; egorova_anna@yahoo.com (A.E.); sofia.shtykalova@gmail.com (S.S.); natashved@mail.ru (N.S.); marianna0204@gmail.com (M.M.); baranov@vb2475.spb.edu (V.B.); 2 Department of Immunology and Intercellular Interactions, D.O. Ott Research Institute of Obstetrics, Gynecology and Reproductology, Mendeleevskaya Line 3, 199034 Saint-Petersburg, Russia; a_selutin@yahoo.com (A.S.); selkovsa@mail.ru (S.S.)

**Keywords:** DNA delivery, peptide-based carriers, gene therapy, thymidine kinase, uterine leiomyoma, integrins, iRGD, pancreatic carcinoma

## Abstract

Uterine leiomyoma (UL) is one of the most common benign tumors in women that often leads to many reproductive complications. Suicide genetherapy was suggested as a promising approach for UL treatment. In the present study, we describe iRGD ligand-conjugated cysteine-rich peptide carrier RGD1-R6 for targeted DNA delivery to αvβ3 integrin-expressing primary UL cells. The physico-chemical properties, cytotoxicity, transfection efficiency and specificity of DNA/RGD1-R6 polyplexes were investigated. TheHSV-1thymidine kinase encoding plasmid delivery to PANC-1pancreatic carcinoma cells and primary UL cells resulted in significant suicide gene therapy effects. Subsequent ganciclovir treatment decreased cells proliferative activity, induced of apoptosis and promoted cells death.The obtained results allow us to concludethatthe developed RGD1-R6 carrier can be considered a promising candidate for suicide gene therapy of uterine leiomyoma.

## 1. Introduction

Uterine leiomyoma (UL) or uterine fibroids, is a benign tumor that develops in the myometrium and is the most common solid tumor for women of reproductive age [1]. It causes subfertility and miscarriages, uterine bleeding, dysfunction of the pelvic organs, pelvic pain and is one of the main reasons for hysterectomy [2]. Current conservative approach for UL treatment—e.g., gonadotropin-releasing hormone agonists (GnRH) therapy—can alleviate the UL symptoms and lead to reduction of tumor volume. However, this treatment is temporary and is associated with severe side effects, primarily with irreversible bone loss [3]. Aromatase inhibitors are successfully used in post-menopausal women, but their prolonged use may also be associated with loss of bone mineral density and increased risk of fractures [4]. Oral GnRH antagonists seem to be promising, but because of high cost and route of administration, they give few advantages over GnRH agonists [5]. In addition, the surgical method continues to be the main method of treatment for this category of patients. However, it strongly affects the childbearing ability for the woman in the long term.

An attractive approach for UL treatment is a localized method that targets specific tumor lesions without interfering with the patient’s fertility or altering hormone levels [5]. The fact that UL is well-localized in the uterus makes it available for using of existing endoscopic techniques. Particularly, it is possible to treat UL by direct intra-tumor drug injection applying ultrasound procedure [6]. UL is a slow-growing tumor with clearly visible clinical manifestations, and it is not necessary to completely remove the fibroid, but only to reduce its size for alleviating symptoms of the disease [7]. All of the above makes UL an attractive target for gene therapy application.

In the field of tumor gene therapy, suicide gene therapy has a considerable attention among the other approaches [8]. Suicide gene therapy is based on the tumor delivery of the “suicidal” gene, most often the thymidine kinase (TK) gene of the herpes simplex virus type 1 (HSV-1), followed by treatment with guanosine analogues (acyclovir or ganciclovir (GCV)) [9,10]. HSV-1 TK, but not mammalian TK, effectively phosphorylates these analogues, which leads to their incorporation into DNA, premature replication termination and as a result induces apoptotic cell death [11]. Importantly, the suicide gene therapy application gives rise to the “bystander effect”, which is that a higher percentage of tumor cell death occurs with a lower percentage of transfected cells [12,13,14]. This phenomenon increases the efficacy of the suicide gene therapy.

Currently, gene therapy does not have enough effective and safe ways for targeted gene delivery. Viral vectors are highly effective in gene delivery and expression [2,15]. However, the main limitations of virus-mediated gene delivery include restricted DNA capacity, toxicity, immunogenicity, cancer development and, in most cases, unaffordable high cost. Non-viral gene delivery vehicles while still showing lower transfection efficiency are developed to overcome the bottlenecks weaknesses of viral vectors and to create artificial virus-mimicking systems [16,17,18,19,20].

Peptide-based gene delivery systems have many advantages over other non-viral ones, including biocompatibility, biodegradability, limited toxicity and large-scale production capabilities [21,22]. Arginine-rich peptides belonging to cationic cell penetrating peptides (CPPs) are effective for in vitro and in vivo gene delivery [23,24,25]. Previous studies have shown that CPPs crosslinked by disulfide bonds promote efficient gene transfection and low cytotoxicity, due to the fact that disulfide bonds can be split by intracellular reducing compounds as glutathione for to release DNA [26,27,28]. Importantly, cysteine-flanked cross-linking peptides (CLP) have a higher charge density, which increases their ability to condense DNA [29]. Interestingly, a higher concentration of glutathione presents exactly in tumor cells compared to normal ones that make the CLP usage promising for tumor gene therapy [30]. Peptides modified with histidine residues can destroy endosomal membrane due to the imidazole protonation facilitating DNA diffusion from endosome to cytosol by proton sponge effect [31,32,33]. By using ligand-modified peptides, DNA can be selectively delivered to the tumors what can reduce associated severe side effects, e.g., unspecific toxicity.

Among the various peptide ligands for targeted tumor therapy, the motif RGD specific to αv-integrins is actively studied. These integrins are overexpressed on the surface of tumor cells, but their expression is lesser in normal tissues [34]. For example, αvβ3 integrins are significantly upregulated in uterine leiomyoma cells as compared to normal myometrium that makes this receptors perspective for targeted gene therapy of UL [35]. Linear RGD and different cyclic RGD-ligands have been developed and actively studied for tumor targeting [2,14,36,37,38,39]. Compared to linear RGD sequences, cyclic ones favor in selectivity and stability and are widely used for strong binding to αv-integrins. Cyclic iRGD ligand acts as both αvβ3 integrin- and neuropilin-1-targeting peptide, and significantly enhances tumor penetration. iRGD ligand exhibits more effective penetration ability and higher accumulation in tumors compared to the most of cyclic RGD ligands [40]. The most recent study demonstrates the crossing of blood-brain-barrier and systemic siRNA delivery to glioblastoma by means of iRGD-modified protein nanoparticles based on polymerized human serum albumin [41].

In the present study, we studied the potency of the peptide carriers for suicide gene therapy of tumor cells, in particular primary uterine leiomyoma cells. For these purposes, we combined iRGD ligand-modified carrier RGD1 and cross-linking arginine-rich R6 peptide carrier to provide more efficient targeted transfection of the uterine leiomyoma cells [29,42]. The physico-chemical properties, transfection efficiency, transfection specificity and cytotoxicity of the obtained vehicles were investigated in detail. The suicide gene therapy of UL was simulated by transferring the HSV-1 TK gene to primary leiomyoma cells obtained from the uterine fibroids after myomectomy. Cells death induced by HSV-1 TK expression with subsequent GCV treatment was assessed by proliferative activity measurement, living cell counting and quantifying of apoptotic cells number. Here, we demonstrate that RGD1-R6 carrier is highly efficient in promoting the tumor’s cell death.

## 2. Materials and Methods 

### 2.1. Cell Lines and Expression of αvβ3 Integrins in Leiomyoma Cells

Human kidney (293T) and human pancreatic (PANC-1) cell lines were obtained from the Cell Collection of the Institute of Cytology RAS (Saint-Petersburg, Russia). Primary leiomyoma cells were obtained after myomectomy in the D.O. Ott Research Institute of Obstetrics, Gynecology and Reproductology (Saint-Petersburg, Russia) as previously reported [43]. Briefly, dissected collagenase IV treated fibroids were resuspended in AmnioMax Basal Medium with 10% AmnioMax Supplement serum (Thermo Fisher Scientific, Carlsbad, CA, USA). The UL cells suspension was transferred to cultural flasks and after the first passage the AmnioMax was substituted by DMEM-F12 with 10% fetal bovine serum (Thermo Fisher Scientific, Carlsbad, CA, USA). The cell culturing was continued for up to 6 weeks at 37 °C with 5% CO_2_.

The expression of αvβ3 integrins in leiomyoma cells was determined by flow cytometry after cells detaching from flasks with 5 mM EDTA in 1× phosphate-buffered saline (PBS) (Rosmedbio, Saint-Petersburg, Russia) and staining with FITC mouse anti-human CD51/CD61 antibodies (BD Pharmingen, San Jose, CA, USA) for 20 min at room temperature [43]. Flow cytometry was conducted using BD FACS-Canto II cytofluorimeter (Becton-Dickinson Biosciences, Franklin Lakes, NJ, USA). A total of 10,000 cells were taken into account.

### 2.2. Peptide and Reporter Plasmids

R_9_H_4_CRGDRGPDC (RGD1), R_9_H_4_ (RGD0) and CHR_6_HC (R6) peptides were synthesized in NPF Verta, LLC (Saint-Petersburg, Russia) and stored as a dry powder at −20 °C as mentioned previously [29,42] (Table 1). Molecular structure of the peptides is presented in Figure S1. RGD0 and R6 carriers were dissolved in dH_2_O at 2 mg/mL and stored at −20 °C. RGD1 was cyclized, evaporated and stored at −70 °C as described in previous studies [42].

The pCMV-lacZ plasmid containing β-galactosidase gene under control of the cytomegalovirus promoter was gifted by Professor B. Sholte, Erasmus University Rotterdam, Netherlands. The pEXPR-IBA5-eGFP plasmid with green fluorescence protein (GFP) gene was obtained from IBA GmbH, Göttingen, Germany. The pPTK1 plasmid containing HSV1 herpes virus thymidine kinase gene was provided by Dr. S.V. Orlov from the Institute of Experimental Medicine, St. Petersburg, Russia. The plasmids were isolated using a Qiagen Plasmid Giga kit (Qiagen, Hilden, Germany) under endotoxin free conditions (Qiagen) and diluted in water to 0.5–1 mg/mL and stored at −20 °C.

### 2.3. Preparation of Carrier/DNA Complexes

RGD1-R6 and RGD0-R6 carriers were obtained by mixing solutions of RGD1 or RGD0 and R6 peptides in equimolar concentrations before the addition of plasmid DNA. DNA/peptide complexes were prepared in Hepes-buffered mannitol (HBM) (5% *w*/*v* mannitol, 5 mM Hepes, pH 7.5) at various N/P ratios by adding peptide to DNA solution as described previously [29]. Complexes were left at room temperature for 2 h for disulphide bonds formation. The charge ratio of polyethyleneimine (branched PEI 25 kDa; Sigma, St. Louis, MO, USA) to DNA was taken as 8/1.

### 2.4. DNA Binding and DNAse I Protection Assays

Peptide binding to DNA was analyzed using the ethidium bromide (EtBr) fluorescence quenching method in a Wallac 1420D scanning multilabel counter (PerkinElmer Wallac Oy, Turku, Finland) at 590 nm emission (540 nm excitation). EtBr displacement was calculated as (F − Ff)/(Fb − Ff), where Ff and Fb are the EtBr fluorescence values in the absence and presence of DNA [44].

For DNAse I protection assay 10 μL of the peptide/DNA complexes was prepared at different N/P ratios and incubated with 0.5 units of DNase I (Ambion, Austin, TX, USA) for 30 min at 37 °C followed by 2 min of DNAse I activation as described previously [44]. DNA was released from complexes with overnight 0.1% trypsin treatment at 37 °C. The DNA integrity was analyzed by 1% agarose gel electrophoresis.

### 2.5. Size and ʐ-Potential Measurement of Peptide/DNA Complexes

The size and zeta potential of the complexes at different charge ratios was determined using dynamic light scattering and microelectrophoresis, respectively. The measurements were performed using zetasizer NANO ZS (Malvern instruments, Malvern, UK) three times independently.

### 2.6. Transmission Electronic Microscopy

Microphotographs of the peptide/DNA complexes at 8/1 and 12/1 charge ratios were obtained using a transmission electron microscope Libra 120 (Carl Zeiss, Oberkochen, Germany). To obtain electron microphotographs, a method of negative staining with a 1% aqueous solution of uranyl acetate was used.

### 2.7. SYBR-Green Exclusion Assay

For monitoring of the SYBR-Green displacement peptide/DNA complexes were prepared as described above with addition of 1× SYBR-Green (Amresco, Solon, OH, USA) at N/P ratio of 8:1. The fluorescence values (excitation 485 nm, emission 590 nm) were continuously monitored for 120 min to determine indirectly the kinetics of cross-linking [44]. Fluorescence was measured on Wallac 1420D scanning multilabel counter. Displacement was calculated as (F − Ff)/(Fb − Ff), where Ff and Fb are the fluorescence intensities of SYBR-Green in the absence and presence of DNA.

### 2.8. Ellman’s Assay

Estimation of disulfide bonds amount in peptides bound to DNA was analyzed directly by using Ellman’s assay [45]. The peptide/DNA complexes were prepared, aliquoted and mixed with solution of 5-5′-dithiobis (2-nitrobenzoic acid) (DTNB or Ellman’s reagent, Sigma, St. Louis, MO, USA) in 0.1 M phosphate buffer (pH 8.0) according to previous description [44]. Absorbance measurements were performed in Multiscan plus P reader (Labsystems, Helsinki, Finland) with wavelength 405 nm. Relative amount of free thiol groups was calculated as (P − Pf)/(Pb − Pf), where Pf and Pb are the absorbance in the absence (free peptide only) and the presence of DNA.

### 2.9. Relaxation of Carrier/DNA Complexes by Dextran-Sulfate and DTT Destabilization

Dextran-sulfate (DS; Sigma, St. Louis, MO, USA) was added to the prepared complexes at three-fold charge excess relative to the peptide. At 0 min and after 24 h of incubation EtBr fluorescence was measured on Wallac 1420D scanning multilabel counter and dye displacement was calculated.

For study of DTT destabilization the peptide/DNA complexes were prepared with addition of 1× SYBR-Green followed by incubation of complexes with 200 mM DTT (Amresco, Ohio, OH, USA) for 1 h at 37 °C. The fluorescence was measured, and SYBR-Green displacement was calculated as described above.

### 2.10. Gene Transfer and Cytotoxicity Assays

A day before experiment, PANC-1 or 293T cells were seeded in 48-well plates at a density of 5.0 × 10^4^ cells per well. Transfections were performed in serum-free medium with 2 µg of DNA per well for 4 hours followed by 48 h incubation in serum-contained medium as described previously [42]. Some transfections were performed under serum-present conditions in fully supplemented medium. After transfection with pCMV-lacZ plasmid, cells were lysed, and β-galactosidase activity (mU) normalized by the total protein concentration in cell extracts was determined as described previously [44]. The β-galactosidase activity was measured on Wallac 1420D scanning multilabel counter (355 nm excitation, 460 nm emission). The total protein concentration of the cell extracts was determined using Bradford reagent (Helicon, Moscow, Russia) in Multiscan plus P reader with wavelength 620 nm. For competition, study free c(RGDfK) peptide with a 10-fold excess was added to PANC-1 cells 15 min before transfection [46]. Percentage of GFP-positive cells was determined by flow cytometry at 48 h after the transfection of PANC-1 cells with pEXPR-IBA5-eGFP plasmid.

The cytotoxicity of DNA/peptide complexes was assessed on PANC-1 cells using Alamar blue reagent (BioSources International, San Diego, CA, USA) after 16 h of incubation with it as described previously [44]. The fluorescence was measured on Wallac 1420D scanning multilabel counter (excitation 544 nm, emission 590 nm) and the relative fluorescence intensity was calculated.

### 2.11. Cellular Uptake of Peptide/DNA Complexes

PANC-1 cells were seeded at a density of 6 × 10^4^ cells/well in 48-well plates. Before formulation of the complexes, DNA was labeled with YOYO-1 iodide (Thermo Fisher Scientific, Waltham, MA, USA) (1 molecule of the dye per 50 base pairs). Transfection was performed as described in subchapter 2.10. After 2 h of the complexes treatment, the cells were washed twice in 1× PBS (pH 7.2) and once with 1 M NaCl (in 1× PBS). Then, the cells were detached, resuspended and incubated with propidium iodide solution (50 µg/mL in 1× PBS) for 15 min in the dark to exclude dead cells. Subsequently, the cells were processed by flow cytometry with a BD FACS-Canto II cytofluorimeter. The results were presented as RFU/cell. 10,000 living cells were taken into account.

### 2.12. Suicide Gene Therapy

PANC-1 or primary leiomyoma cells were seeded on 96-well plates (“Nunc”) at 1.5 × 10^4^ cells per well and cultured for 24 h. Transfections were performed in serum-free medium with 0.7 µg of DNA (pPTK1 plasmid) per well. The plates were incubated for 2 h and the medium was changed to standard one for the next 24 h. After that, the medium was also changed to standard one but to containing 50 μg/mL of ganciclovir and the plate was left for 24 or 96 h. 

For proliferation activity measurement 96 h later, the medium was replaced for the fresh one containing 10% Alamar Blue solution, and the plate was incubated for another 2 h. Fluorescence intensity was measured on a Wallac 1420D fluorimeter at wavelengths 530/590 nm. The number of living cells was calculated as (F − Ff)/(Fb − Ff), where Fb and Ff are the fluorescence intensities in untreated control and without cells, respectively. 

Photographs of cells were obtained on a microscope AxioObserver Z1 (Carl Zeiss, Oberkochen, Germany) using the AxioVision program at 100× magnification.

For counting the living cells number, the Trypan blue dye exclusion method was used. After 96 h of incubation cells were harvested with Trypsin-EDTA (Thermo Fisher Scientific, Carlsbad, CA, USA) 0.25% followed by addition of 0.4% Trypan blue solution (Sigma-Aldrich, Munich, Germany) at a 1:1 volume ratio, the unstained cells were counted using a hemocytometer (MiniMedProm, Dyatkovo, Russia).

To quantify the relative amount of apoptotic and necrotic cells the ApoDETECTannexin V-FITC kit (Invitrogen, Darmstadt, Germany) was used according to manufacturer’s recommendations after 24 h of incubation. The cells were analyzed by BD FACS-Canto II cytofluorimeter.

### 2.13. Statistical Analysis

Statistically significant differences were analyzed by the Mann–Whitney U-test and by Student’s *t*-test, using Instat 3.0 (GraphPad Software Inc., San Diego, CA, USA). *p* < 0.05 was considered statistically significant.

## 3. Results and Discussion

### 3.1. Carrier Design

Herein, we report the study of RGD1-R6 carrier, developed by combination of iRGD ligand-modified RGD1 carrier with cysteine-flanked R6 peptide [29,42]. The specific binding of RGD ligand to integrin αvβ3 offers a promising strategy aimed at the delivery of therapeutic molecules to tumor cells [34]. The tumor-penetrating iRGD peptide not only has a high affinity to integrin αvβ3 but also helps to reach the depth of tumors by subsequent binding to neuropilin-1 [40]. In our previous study we found that the cell penetration and transfection efficiency of the iRGD-peptide/DNA complexes greatly depended on the amount of ligand part in the polyplexes composition. In fact, it is necessary that at least 50% of the polyplexes must be modified with the iRGD ligand to provide αvβ3-targeted gene delivery [42]. Influence of the RGD ligand amount on the targeting behavior of PEI-PEG copolymer carriers was also described previously [38]. Disulfide cross-linking can contribute to the stable NA-complexes formation followed by NA release in the reducing intracellular environment [47]. Proposed arginine-histidine-cysteine based peptide systems for efficient cellular uptake, endosomal escape and stable complexes already are actively used for in vivo studies [48,49,50,51]. Here, we combined RGD1 and R6 peptides in equimolar concentration to achieve the necessary effects. Unmodified RGD0-R6 carrier was used as a control one. The carriers are presented in the Table 1.

### 3.2. DNA Binding and DNA Protection Properties of the Carriers

**Figure 1:**
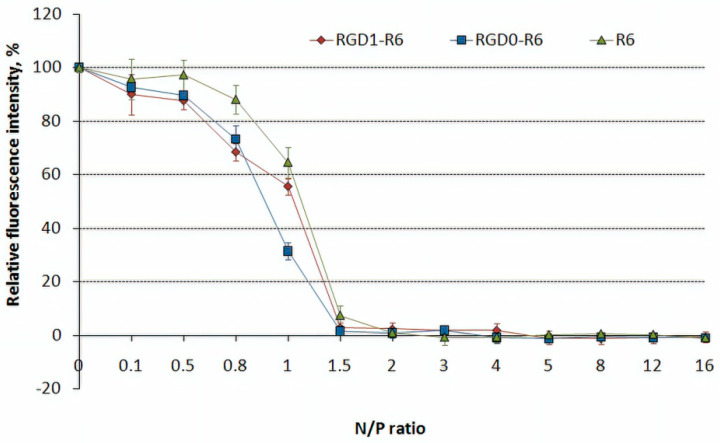
EtBr exclusion assay of DNA complexes with RGD1-R6, RGD0-R6 and R6 carriers. Values are the mean ± SD of the mean of triplicates.

The DNA condensation with RGD1-R6 and RGD0-R6 carriers was determined using the EtBr exclusion assay. R6 peptide only was used as a control one. As shown in Figure 1, the EtBr fluorescence intensity of DNA complexes decreased substantially at an N/P ratio of 1.5/1 (up to 2–7%) compared with that of naked DNA (100%). Thus, the addition of R6 peptide to the composition of the carriers resulted in better DNA condensing properties compared to RGD1 and RGD0 carriers, which can completely condense DNA at N/P ratio 2/1 [42].

**Figure 2:**
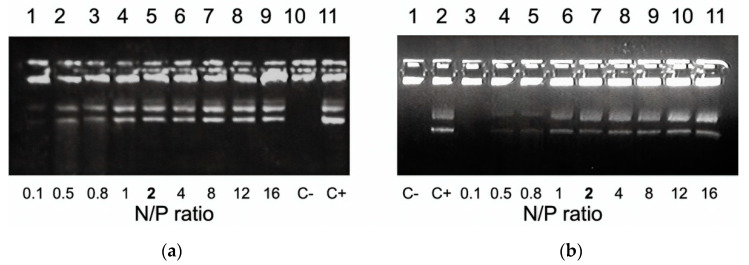
DNase I protection assay of DNA-complexes formed with (**a**) RGD1-R6 and (**b**) RGD0-R6 carriers. N/P ratio in **bold** indicates the beginning of DNA protection. C−, ‘naked’ plasmid DNA treated with DNase I, C+, untreated plasmid DNA.

DNA compaction level directly correlates with its sensitivity to nucleases [52]. We estimated DNA integrity by means of a DNase I protection assay. Naked DNA incubated with DNase I was not detected because of degradation. An increase in the numberof peptides in the complexes resulted in a better DNA protection. Studied carriers were able to protect DNA from nuclease degradation at N/P ratio 2/1 (Figure 2). DNA protective ability of the carriers was comparable with that of arginine-rich R6 peptide and RGD1 carrier [29,42].

The results obtained demonstrate that an uncharged iRGD ligand does not affect the DNA binding and protection abilities. Similar results were previously obtained for RGD1 carrier modified with iRGD ligand and for bPEI modified with cRGD ligand [38,42]. It can be suggested that non-participation of iRGD ligand in DNA binding and protection enable its successful targeting of αvβ3-positive tumor cells.

### 3.3. Kinetics of Carrier/DNA Complexation and Disulfide Bonds Formation during Template Polymerization

Disulfide cross-linking can play an important role in the DNA complexation by cysteine-flanked carriers. R6 peptide moiety in the carrier’s composition forms disulfide cross-links by oxidation of thiol groups in cysteines. Disulfide bonds formation was estimated indirectly by monitoring of DNA complexation kinetics and directly by determination of free thiol groups amount during 2 h of DNA binding experiment. 

**Figure 3:**
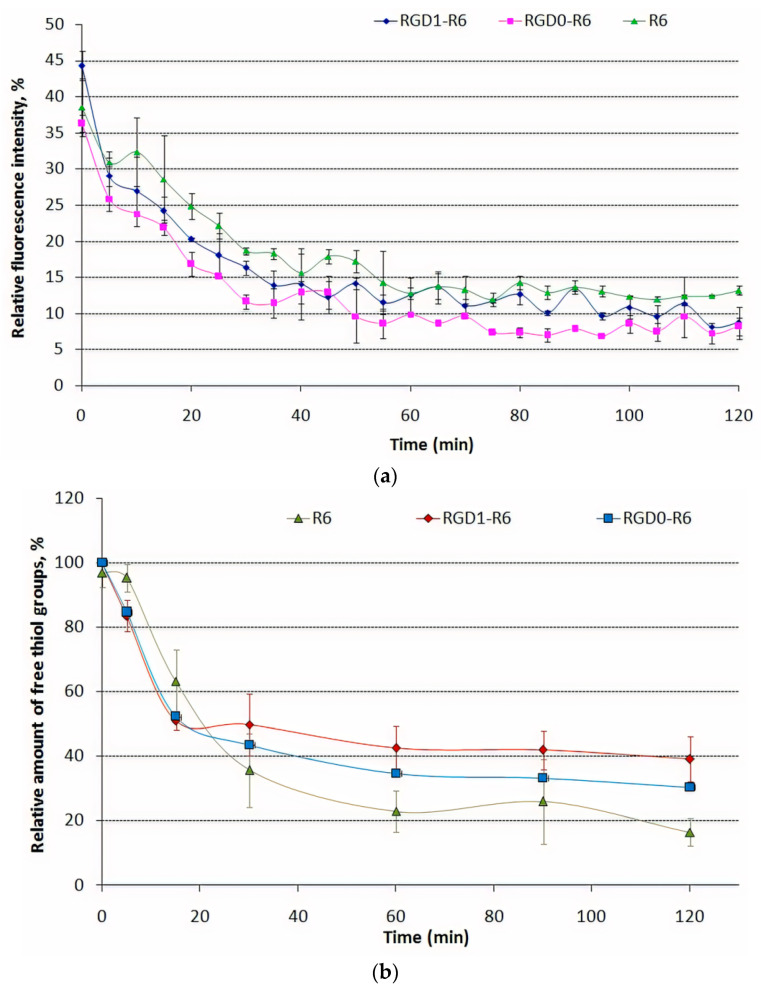
Monitoring of the polyplexes formation: (**a**) by SYBR Green exclusion assay and (**b**) by Ellman’s assay to measure of relative amount offree thiol groups inRGD1-R6/DNA, RGD0-R6/DNA and R6/DNA complexes. Values are the mean ± SD of the mean of triplicates.

The template polymerization kinetics of RGD1-R6, RGD0-R6 peptide carriers was studied via SYBR-Green exclusion assay. SYBR-Green dye was used because of its greater DNA affinity and higher fluorescence intensity even when bind to DNA in polyplexes at high N/P ratios. The kinetics of DNA complexation by RGD1-R6 and RGD0-R6 carriers was compared with that of R6/DNA polyplexes at 8/1 charge ratio. The addition of the carriers to DNA immediately before the incubation resulted in a slow decrease in SYBR-Green fluorescence intensity during the incubation period until 8–13% of fluorescence intensity of stained free DNA (Figure 3a). A gradual decrease in fluorescence intensity indirectly indicates the time-dependent formation of tight DNA-complexes due to disulfide cross-linking. On the other hand, DNA complexation by non-reducible carriers results in constant SYBR-Green fluorescence during whole the incubation time [29].

The results above were proved using Ellman’s assay (Figure 3b). As expected, a clear relationship between the SYBR Green quenching and free thiol amount was found. The relative amount of the remaining thiol groups in the R6/DNA complexes at an N/P ratio of 8/1 decreased relatively rapidly over time and reached 16% after 2 h. The reduction of thiol groups in RGD1-R6 and RGD0-R6 carriers also was determined over time, but was different from those found in R6/DNA complexes (about 30–39% free thiol remained). We suggest that incomplete formation of interpeptide disulfide bonds in RGD1-R6 and RGD0-R6 carriers can be explained by competition in DNA condensation between RGD1/RGD0 and R6 peptides. According to the condensation theory the condensing counterions can move freely along the polyion backbone and cationic cysteine-flanked R6 peptides can bind each other with high efficiency during DNA template condensation [29,53]. However, in the presence of competitive cationic RGD1 or RGD0 peptides the movement of R6 peptides may be hindered and some thiol groups remain unreacted. Thus, we can assume that competition of arginine-containing RGD1/RGD0 and R6 peptides for DNA binding may influence on time and efficacy of disulfide bonds formation. The influence of DNA complexation stereochemistry on the efficiency of disulfide bonds formation was previously demonstrated for lysine- and arginine-rich peptides [29].

### 3.4. DNA Release after DTT and DS Treatment of the Polyplexes

**Figure 4:**
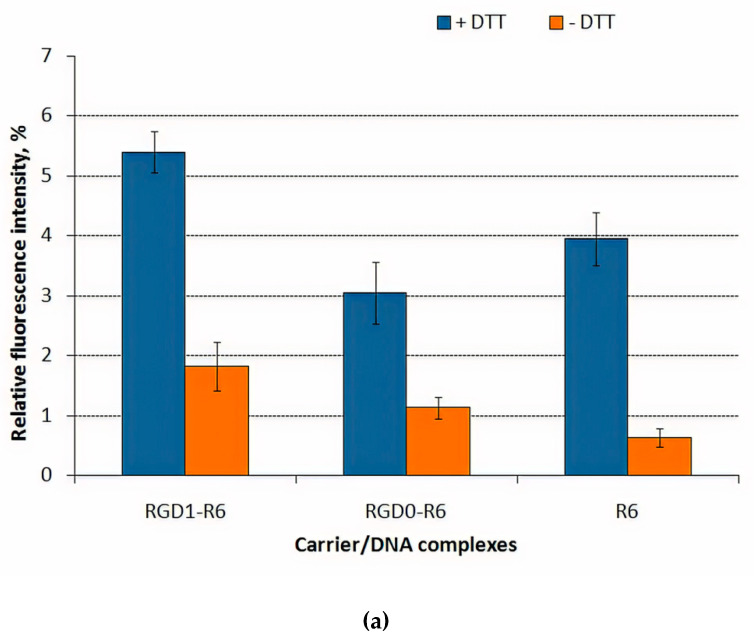

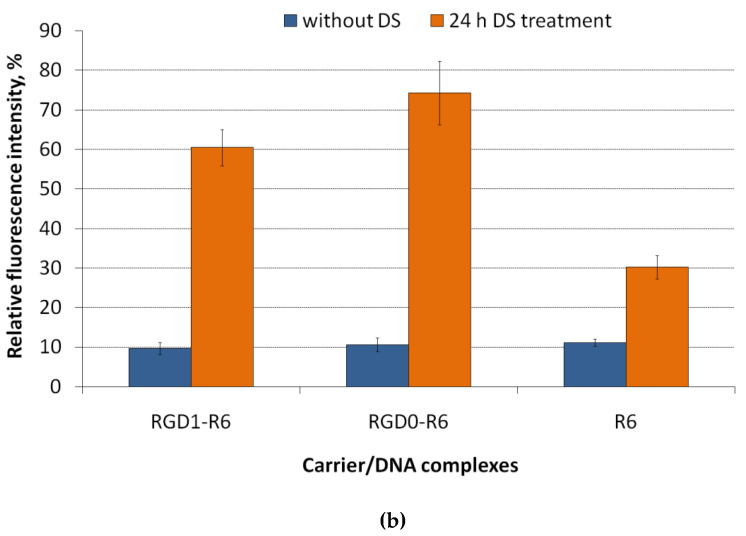
DNA release (**a**) after DTT treatment of DNA-complexes with RGD1-R6, RGD0-R6 and R6 carriers and (**b**) after relaxation of DNA complexes with RGD1-R6, RGD0-R6 and R6 carriers after 24 h of DS treatment in three-fold charge excess. Values are the mean ± SD of the mean of triplicates.

To prove the role of the disulphide bonds in the formation of RGD1-R6/DNA and RGD0-R6/DNA complexes, we destabilize them by dithiotreitol (DTT), which is a typical reducible agent. DTT treatment was performed for DNA-complexes at 8/1 charge ratio (Figure 4a). Before DTT treatment, we observed the complete DNA condensation for all the tested polyplexes. After 1 h incubation of the complexes with DTT, a partial DNA release for complexes was detected. DTT treatment resulted in 2–5-fold increase in fluorescence intensity what reflects DNA release from the complexes. The values of the relative fluorescence after incubation with DTT compared to free DNA (100%) indicate a not very strong effect of disulfide bonds on the stability of polyplexes in comparison with electrostatic forces associated with the tight DNA packaging by oligoarginine [29]. Additional data on the stability of DNA-polyplexes were obtained after dextran-sulfate treatment (Figure 4b).

Interaction of negatively-charged GAGs (heparan sulfate, chondroitin sulfate B and C etc.) with polyplexes may influence on DNA transfer. GAGs are found both within cells and in extracellular space [54]. These anionic components interaction with polyplexes may reduce DNA delivery via complexes inhibition and/or DNA decompactization [55,56]. On the other hand, the ability of carriers to release DNA from complexes inside the cells for subsequent expression is an important parameter that can promote the transfection process [57]. In addition, expression of some sulfated GAGs on the cell surface may also play a positive role in gene delivery [58]. The RGD1-R6/DNA and RGD0-R6/DNA complexes were found to be susceptible to DS and were easily relaxed by 3-fold charge excess of dextran-sulfate. However, R6/DNA complexes demonstrate increased resistance against polyanions that may be the result not only of tight DNA packing by oligoarginines but also an increased amount of disulphide bonds compared to RGD1-R6 and RGD0-R6 carrier/DNA complexes.

### 3.5. Size and ʐ-Potential of the Carrier/DNA Complexes

**Figure 5:**
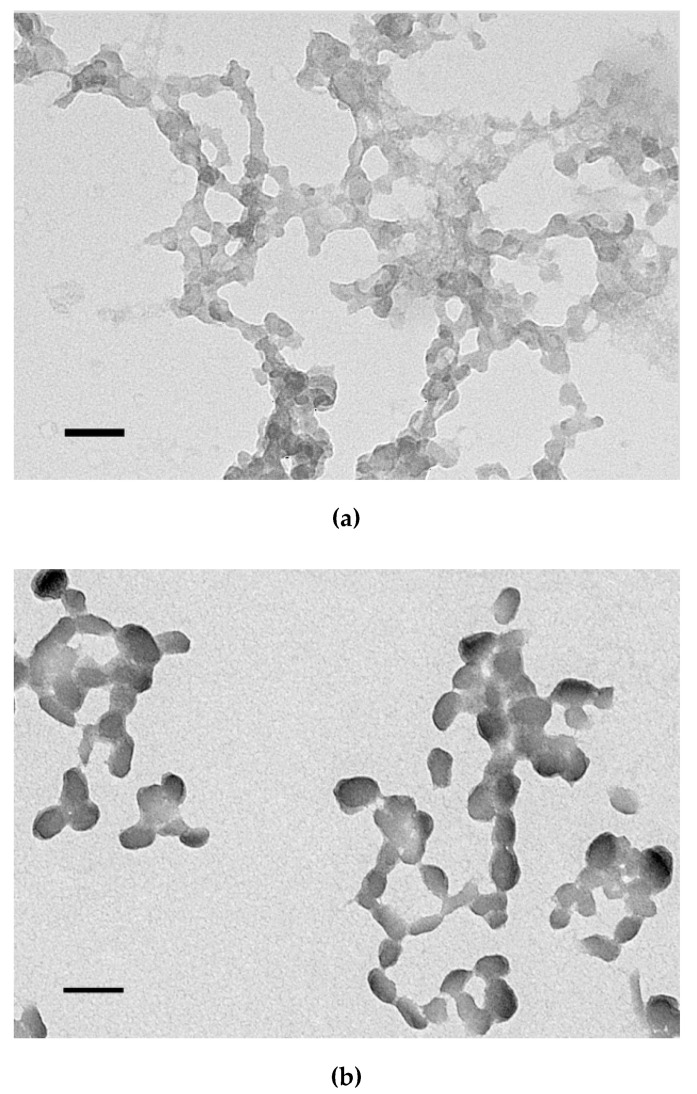

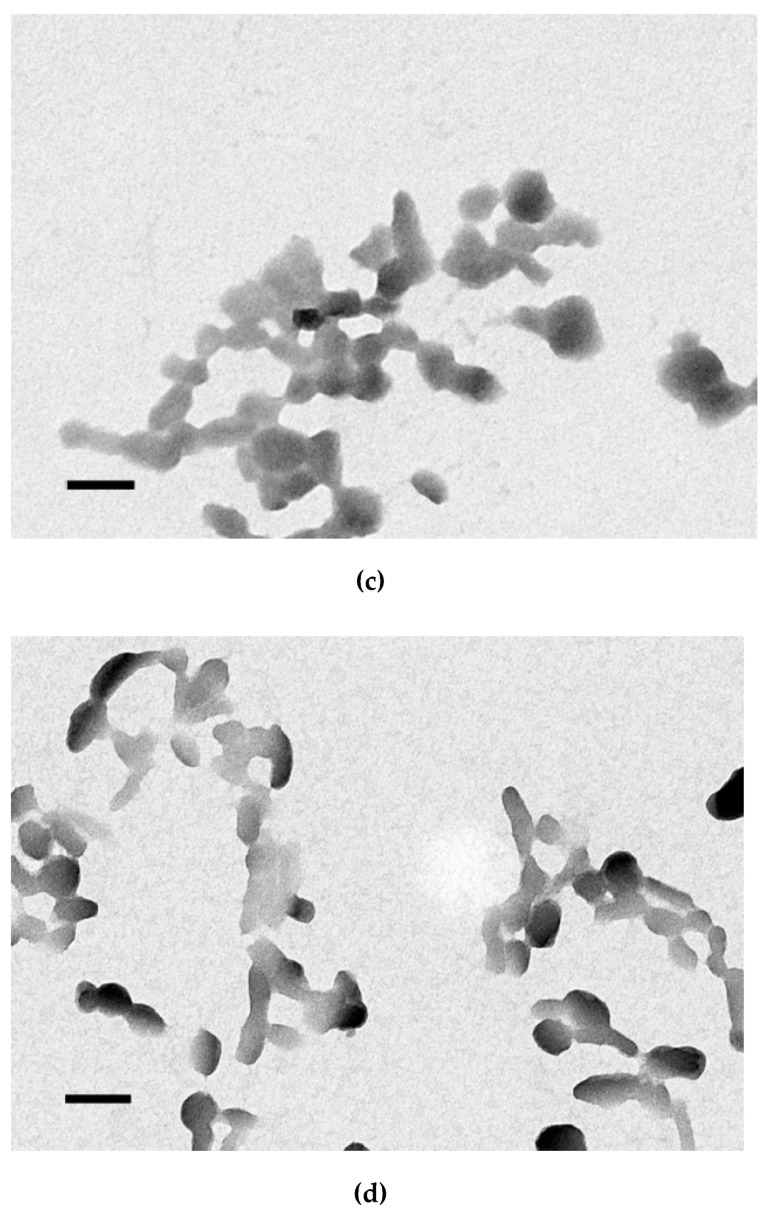
Typical microphotographs of the carrier/DNA complexes obtained by transmission electron microscopy: (**a**,**b**) RGD1-R6-polyplexes and (**c**,**d**) RGD0-R6-polyplexes formed at N/P ratios 8/1 and 12/1, respectively. The scale bar corresponds to 100 nm.

The size and charge of the polyplexes are important parameters determining the internalization pathway and a success of gene transfer [59,60]. The size of the carrier/DNA complexes was found in the range 100–130 nm with exception of polyplexes formed at N/P ratio 4/1 which size exceeds 300 nm (Table 2). In this case, the presence of uncharged ligand part to the carrier composition does not affect the size of polyplexes. Smaller polyplexes could enter the cell by clathrin-mediated endocytosis, which is characteristic of particles with a diameter of 100–150 nm [61]. Further, the smallest polyplexes formed at N/P ratios 8/1 and 12/1 were studied by transmission electron microscopy. According to electronic microphotographs, the size of the studied polyplexes corresponds to data obtained using dynamic light scattering. The polyplexes have globular shapes and do not tend to aggregate (Figure 5). The zeta-potential of polyplexes is optimized for effective electrostatic interaction with the plasma membrane [59]. The zeta potential of studied DNA complexes was positive and ranged in 16–26 mV at N/P ratios 8/1–16/1 and 4–5 mV for polyplexes formed at N/P ratio 4/1 (Table 2). Complexes with a positive zeta potential were shown to have a higher transfection efficiency compared to their negatively charged counterparts [62]. The surface of the positively charged complexes could facilitate the cell penetration due to interaction with negatively charged components of the membrane.

### 3.6. Cytotoxicity of DNA-Polyplexes

**Figure 6:**
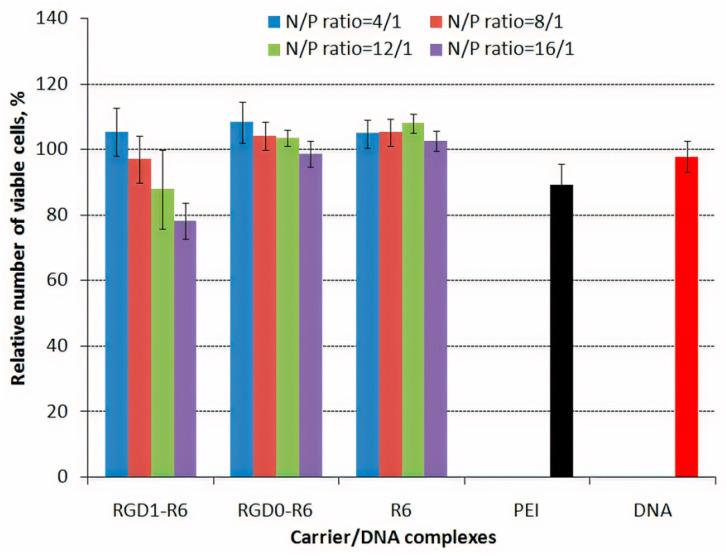
The cytotoxicity after transfection of DNA complexes with RGD1-R6, RGD0-R6 and R6 carriers. Values are the mean ± SD of the mean of triplicates.

Polycationic gene delivery systems could be cytotoxic due to their molecular weight, positive charge etc. [63]. Reducing of cytotoxicity is an important step towards to development of safe gene delivery system. The inclusion of the disulfide bridges to polymers for their biodegradability can both decrease cytotoxicity and improve the transfection efficiency [64]. Cytotoxicity of the studied carrier/DNA complexes was performed using Alamar Blue assay (Figure 6). PANC-1 cells were transfected with complexes of RGD1-R6, RGD0-R6, R6 and pCMV-lacZ plasmid at N/P ratios of 4/1–16/1. Integrins αvβ3 were previously shown to be overexpressed on this cell line (34.5% of the cells) [42].

Alamar Blue assay demonstrated that the most of DNA-complexes at studied N/P ratios did not show significant cytotoxicity, indicating that these polyplexes are consisting of non-toxic DNA carriers. The relative number of viable cells after treatment with these complexes was comparable to that of naked DNA and was higher compared to non-reducible PEI/DNA polyplexes. The only exception is RGD1-R6-polyplexes formed at high charge ratio 16/1 which were more toxic than ligand-free RGD0-R6-polyplexes and showed similar to PEI-polyplexes level of cytotoxicity (Figure 6). This fact allows us to suggest that the observed toxicity can be a result of iRGD ligand-mediated cells detachment from the substrate. Previously, it was demonstrated that excessive RGD ligands binding to the adhesion receptors can disturb the cell cycle and metabolism processes [65].

### 3.7. Gene Transfer

**Figure 7:**
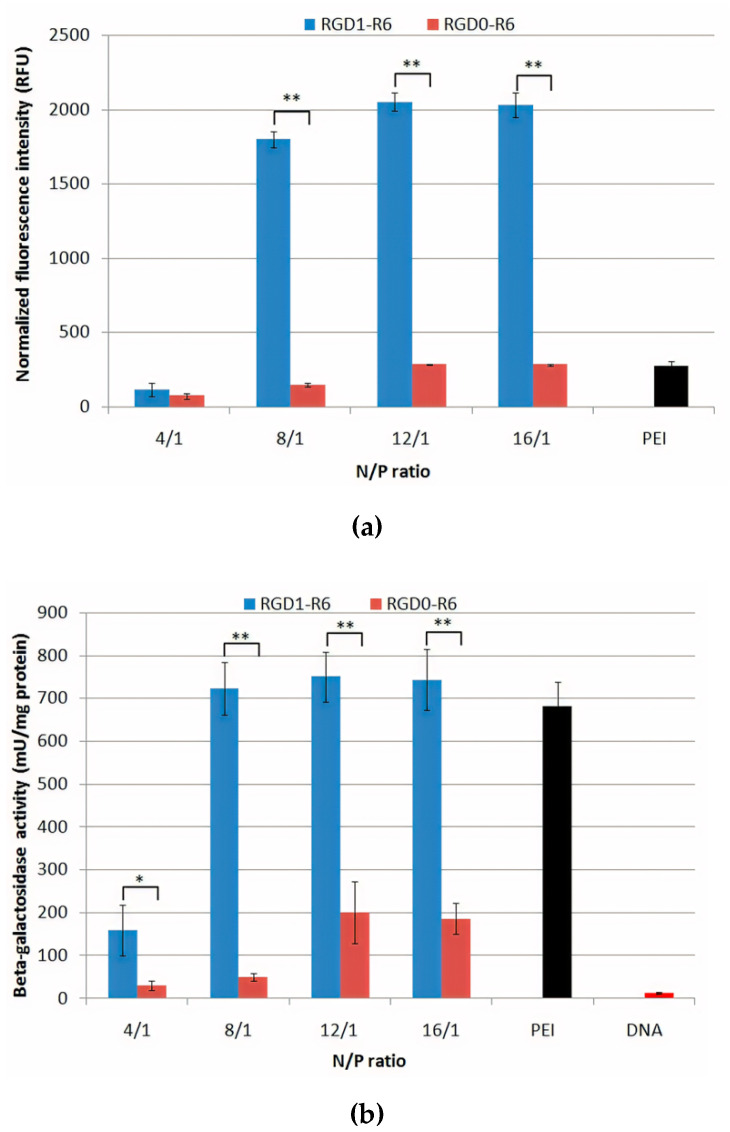

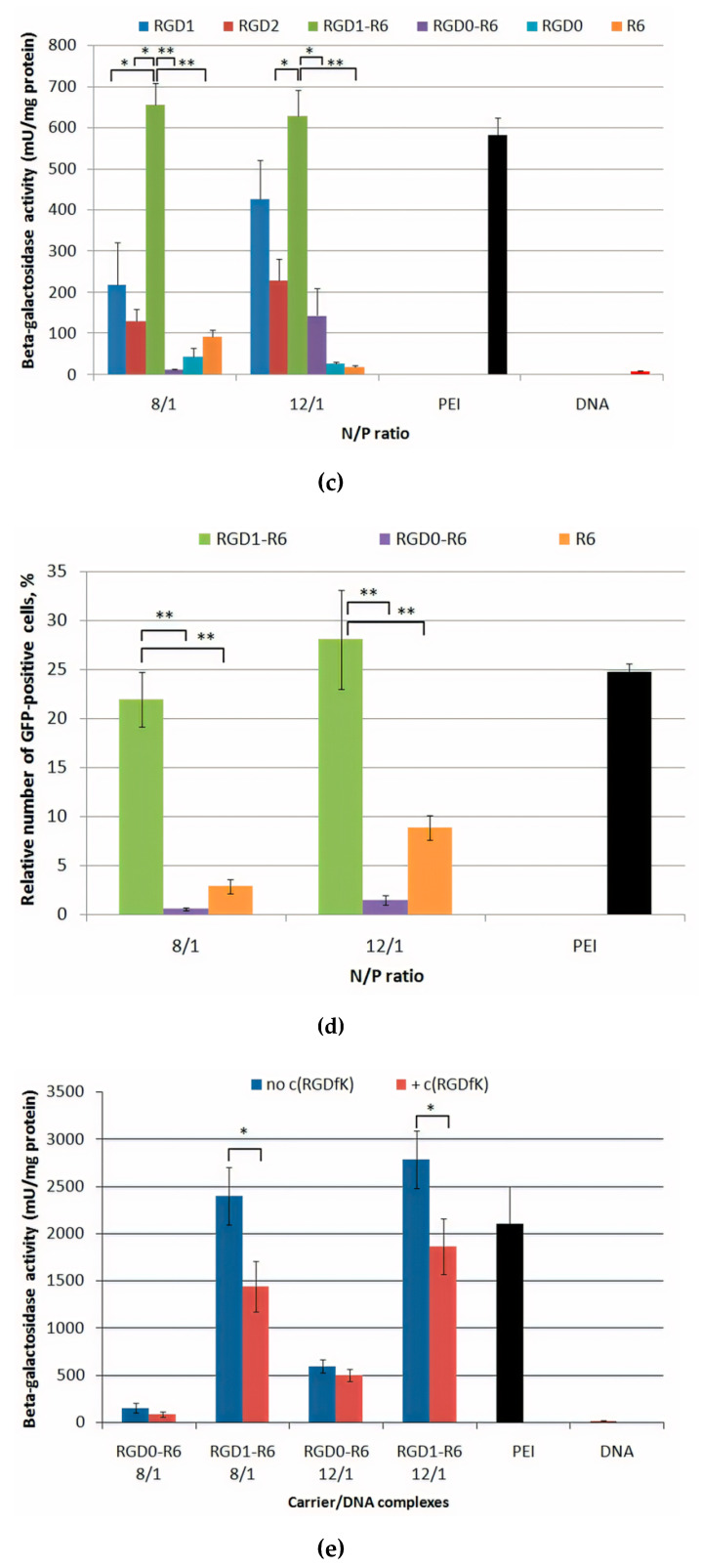

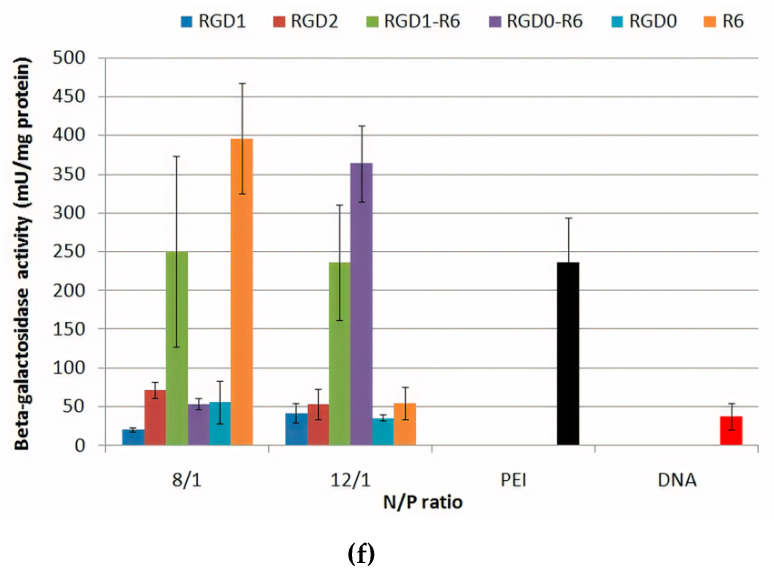
Uptake and transfection efficacy evaluation of RGD1-R6 and RGD0-R6-polyplexes in PANC-1 cells using (**a**) YOYO-1-labeled pDNA, (**b**,**c**) pCMV-lacZ plasmid and (**d**) pEXPR-IBA5-eGFP plasmid; (**e**) competition assay with free cyclic (RGDfK) peptide and RGD1-R6/DNA or RGD0-R6/DNA complexes at N/P ratios of 8/1 and 12/1 in PANC-1 cells; (**f**) transfection efficacy evaluation of RGD1-R6 and RGD0-R6-polyplexes in 293T cells using pCMV-lacZ plasmid. Values are the mean ± SD of the mean of triplicates. * *p* < 0.05, ** *p* < 0.01—compared to the complexes as indicated.

Uptake and transfection experiments were performed using PANC-1 cells that overexpress αvβ3 integrins and thus, previously, have been used to study RGD-specific targeting and transfection efficiency [42]. For cellular uptake efficacy evaluation, we used polyplexes formed at 4/1–16/1 charge ratios with fluorescently-labeled DNA. An increase innormalized fluorescence intensity in the analyzed cells indicated successful uptake of the studied DNA-complexes. It can be seen that transfection with RGD1-R6/DNA polyplexes formed at high charge ratios (8/1–16/1) resulted in a significant increase inthe cellular fluorescence in comparison with control RGD0-R6/DNA and PEI/DNA polyplexes (Figure 7a). Further, the same polyplexes formed with pCMV-lacZ plasmid were used in the transfection experiment. We found that the transfection efficacy of RGD1-R6/DNA polyplexes was significantly higher than that of RGD0-R6/DNA polyplexes and reached a plateau starting from N/P ratio 8/1 (Figure 7b). Similar results were obtained after transfection of PANC-1 cells in presence of 10% fetal bovine serum. It should be noted that the overall level of β-galactosidase activity dropped significantly, suggesting that the studied polyplexes are sensitive to interaction with polyanionic components of serum as it was also shown by DS treatment of the polyplexes (Figure S2 and Figure 4b). However, a relationship found between transfection efficacy and the ligand modification of RGD1-R6-polyplexes was fully reproduced under serum-present transfection conditions. Thus, we concluded that RGD1-R6-polyplexes formed at N/P of 4/1 inefficiently deliver DNA to the PANC-1 cells and can be excluded from further experiments along with the polyplexes formed at N/P of 16/1 which exhibited cytotoxicity (Figure 6). Hereafter, we evaluated the transfection efficacy of DNA-complexes with RGD1-R6 carrier and control carriers RGD0-R6 and R6 formed at N/P of 8/1 and 12/1 (Figure 7c). As comparison we used previously described non-reducible carriers: RGD1 (100 mol% of ligand content), RGD2 carrier (50 mol% of ligand content) and RGD0 (0 mol% of ligand content) [42]. The studied iRGD-modified carriers were more effective in DNA delivery than the unmodified ligand-free carriers. The transfection efficiency of RGD1-R6 carrier was 3–5-fold higher than that of RGD2 peptide with the same amount of ligand and 1.5–3-fold higher than that of fully modified carrier RGD1. Moreover, the efficacy of RGD1-R6 carrier was 5–50-fold higher than that of unmodified carrier RGD0-R6 and cross-linking peptide R6. Also, transfection efficacy of RGD1-R6 carrier was comparable or in some cases even higher that of PEI. The results obtained were additionally confirmed using polyplexes formed with pEXPR-IBA5-eGFP plasmid (Figure 7d). The RGD1-R6/DNA polyplexes mediated transfection resulted in 22% GFP-expressing cells at N/P of 8/1 and 28%—at 12/1 charge ratio, respectively, compared to 6.3 and 17.2% of GFP-positive cells after DNA delivery by RGD1-polyplexes with corresponding N/P ratios [42]. Moreover, the efficacy of RGD1-R6 was comparable and even slightly higher than that of PEI-complexes and much higher than that of RGD0-R6 and R6 ligand-free carriers (0.6–1.5% for RGD0-R6 and 3–9% for R6). These results demonstrate that RGD1-R6 carrier is highly effective for DNA delivery in αvβ3-positive PANC-1 cells. Moreover, the results indirectly confirm RGD-targeted gene delivery. The results obtained are consistent with data from other studies on gene delivery using RGD ligands [42,66,67].

To additionally prove the specificity of iRGD ligand in RGD1-R6 carrier for targeted gene delivery, the inhibitory effect of free competing cyclic RGD was assessed in cell transfection studies. We performed competitive transfection experiments in PANC-1 cells with RGD1-R6/DNA complexes at N/P ratios of 8/1 and 12/1 in the presence of a 10-fold excess of free c(RGDfK) peptide (Figure 7e). Cells pre-treatment with free RGD ligand resulted in 40% decrease inRGD1-R6/DNA polyplexes efficiency. However, the transfection efficiency of RGD0-R6/DNA polyplexes was not affected by the free ligand.Thus, the iRGD ligand in the carrier composition is involved in the complexes internalization via αvβ3 integrins.

The transfection efficiency of RGD1-R6-complexes did not drop to zero after competition study. This may be due to the fact that polyplexes can electrostatically interact with membrane and enter cells via absorptive endocytosis, that can explain inability to completely block the overall high transfection efficiency of RGD1-R6 carrier. In order to further demonstrate targeted gene delivery, we used αvβ3-negative 293T cell line. Previously, we showed that the transfection efficiency of non-cross-linking RGD1 and RGD2 carriers in 293T cells is comparable to control RGD0 [42]. Here, we demonstrate that RGD1-R6 carrier can mediate efficient transfection comparable with PEI. At N/P ratio of 12/1 the efficacy of RGD1-R6 did not differ from that of RGD0-R6 (Figure 7f). Nonetheless, RGD1-R6 carrier at 8/1 charge ratio was more efficient than unmodified one. However, its efficiency was similar to R6 cross-linking peptide. Thus, all the obtained results demonstratedthe specificity and high efficiency of RGD1-R6 carrier. Further, RGD1-R6-based polyplexes at optimal charge ratios were used in suicide gene therapy experiments in PANC-1 and primary leiomyoma cells.

### 3.8. Therapeutic Effect of RGD1-R6/pPTK1 Polyplexes after Ganciclovir (GSV) Treatment

Treatment of uterine leiomyoma continues to be controversial due to lack of effective, non-surgical and localized methods. The precise localization of uterine fibroids and their availability to various endoscopic methods make the disease a promising target for gene therapy application. Today, the ULs gene therapy is at the stage of the developing of effective approaches and systems for gene delivery, while some of the developments demonstrate the successful application in in vitro and in vivo studies [68]. Previously, it has been shown that suicide gene delivery for UL treatment results in significant reduction of tumor size through the induction of apoptosis in both transfected and neighboring tumor cells due to the “bystander effect”. HSV thymidine kinase (HSV TK)-GCV system is one of the most efficient approaches to causing cell death in rapidly dividing cells; in addition, it has a proven efficiency for treatment of many types of tumors [8,69,70]. Moreover, connexins overexpression in UL cells compared to the adjacent normal myometrium can allow avoiding negative suicide effects on healthy myometrium [71].

The main goal of our work was to evaluate the efficacy of suicide gene therapy mediated by RGD1-R6/DNA polyplexes in primary leiomyoma cells. The primary cells obtained from patients after myomectomy can be used as an adequate model for UL gene therapy studies. In order to ensure targeted DNA delivery to UL cells the flow cytometry analysis was used to confirm the presence of αvβ3 integrins on the surface of UL cells. Our results were in agreement with data, which indicated these integrins overexpression on the surface of leiomyoma cells. In total, 73% of UL cells were received to be αvβ3-positive (data not shown).

The suicide effect of RGD1-R6/pPTK1 polyplexes at N/P ratios of 8/1 and 12/1 was evaluated in UL and PANC-1 cells after GCV treatment followed by assessment of the cells proliferative activity using Alamar blue dye or quantifying of the apoptotic and necrotic cells number using ApoDETECT annexin V-FITC kit. For a visual representation of the results, cells were photographed under microscope. The RGD1-R6/pCMV-lacZ polyplexes were used as controls to eliminate the cytotoxicity caused by the polyplexes themselves. Naked plasmid DNAs and cells treated with GCV were also used in the study as comparison controls. It should be noted that all the transfection experiments were performed in serum-free conditions; however, it is generally accepted that serum-present transfection conditions are physiologically more relevant, especially for systemic DNA delivery. In the case of future UL gene therapy, it should be pointed out that the precise localization of uterine fibroids and their accessibility by various endoscopic methods make the disease a promising target for the direct delivery of nucleic acid therapeutics [6]. Thus, at present, we prefer not to treat the polyplexes with FBS during transfection to avoid any possible interference by serum components. Previously, we have shown that direct intra-tissue injection of the peptide-based polyplexes can result in successful nucleic acid delivery [51].

**Figure 8:**
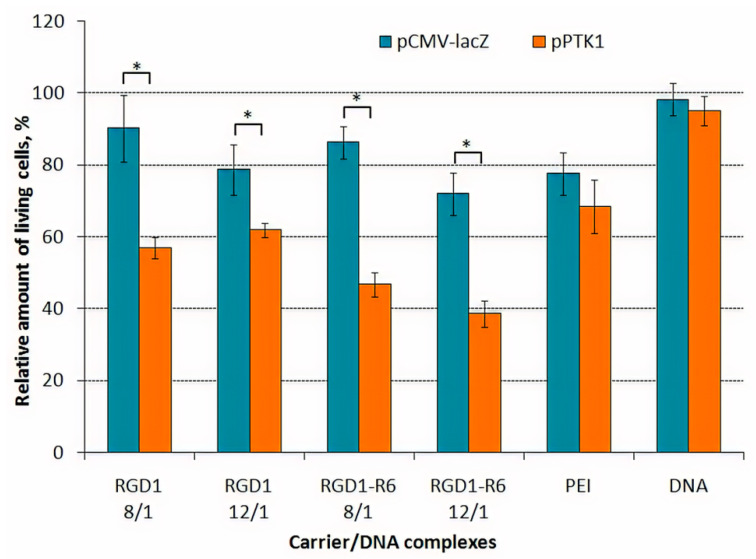
UL cells viability after HSV thymidine kinase expression and GCV treatment. Values are the mean ± SD of the mean of triplicates. * *p* < 0.05 compared to pCMV-lacZ-complexes.

The effect of suicide gene therapy by means of RGD1-R6/pPTK complexes was demonstrated after 4 days of GCV treatment as compared to RGD1-R6/pCMV-lacZ polyplexes. We observed 1.9-fold decrease in UL cells proliferative activity after transfection with RGD1-R6/pPTK complexes compared to RGD1-R6/pCMV-lacZ polyplexes and 1.3–1.6-fold decrease in case of non-cross-linked RGD1/pPTK polyplexes (Figure 8). The cell viability after suicide gene therapy with RGD1-R6/pPTK polyplexes was decreased by 46% compared to the control RGD1-R6/pCMV-lacZ complexes. The cytotoxicity caused by pCMV-lacZ-bearing complexes did not exceed that of PEI-polyplexes. Moreover, PEI/pPTK1 complexes delivery did not lead to changes in the cell proliferation level.

**Figure 9:**
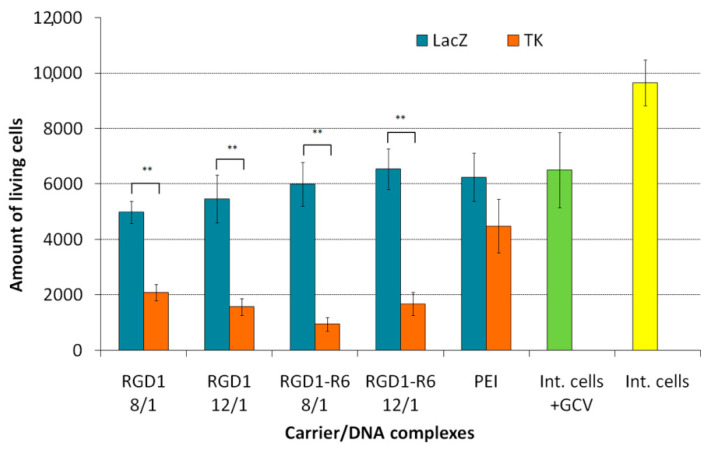
Amount of living UL cells after HSV thymidine kinase expression and GCV treatment. Values are the mean ± SD of the mean of triplicates. ** *p* < 0.01 compared to pCMV-lacZ-complexes.

Similar findings were registered by the Trypan blue method allowing dead cells exclusion from the counting (Figure 9). The number of living cells after transfection by pCMV-lacZ-complexes did not differ from that of cells treated with GCV only. The number of viable UL cells transfected with RGD1-R6/pPTK complexes was significantly decreased up to 16–27% compared to pCMV-lacZ polyplexes. RGD1/pPTK complexes transfection also led to suicide effect resulted in reduced number of viable cells (30–42% compared to control polyplexes). PEI/pPTK1 complexes delivery led to a 1.4-fold decrease in the amount of living cells. Importantly, the suicide effects were more pronounced using Trypan blue method rather than Alamar Blue assay.

Similarly, we registered a 3–4-fold decrease in the PANC-1 cells proliferative activity after pPTK plasmid delivery with RGD1-R6 carrier (Figure S3). For comparison, non-cross-linked RGD1/pPTK polyplexes decreased PANC-1 cells viability only 1.25–1.6-fold [42]. We also observed that the anti-proliferative efficacy of RGD1-R6-based polyplexes was higher than that of PEI/DNA complexes (1.7-fold decrease). The relative number of living cells after suicide gene therapy with RGD1-R6/pPTK polyplexes was reduced by 70% compared to their initial number which was greater than the transfection efficiency (22–28% of PANC-1 cells transfected) (Figure S3).

**Figure 10:**
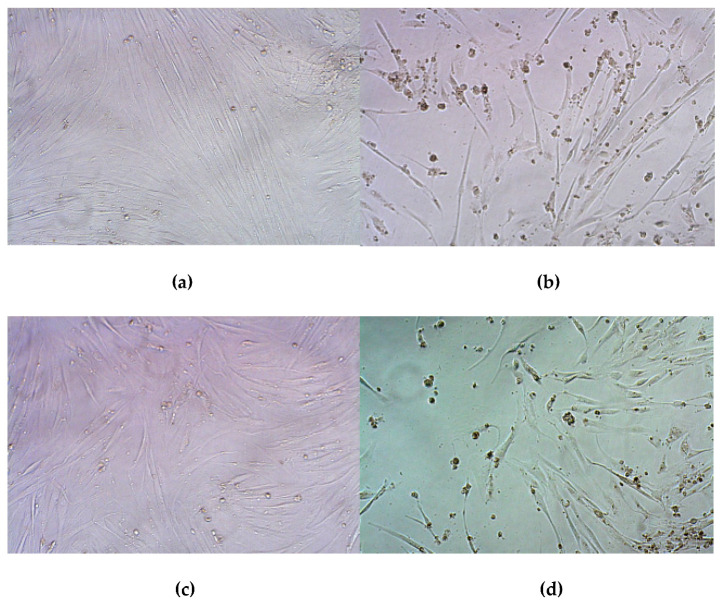

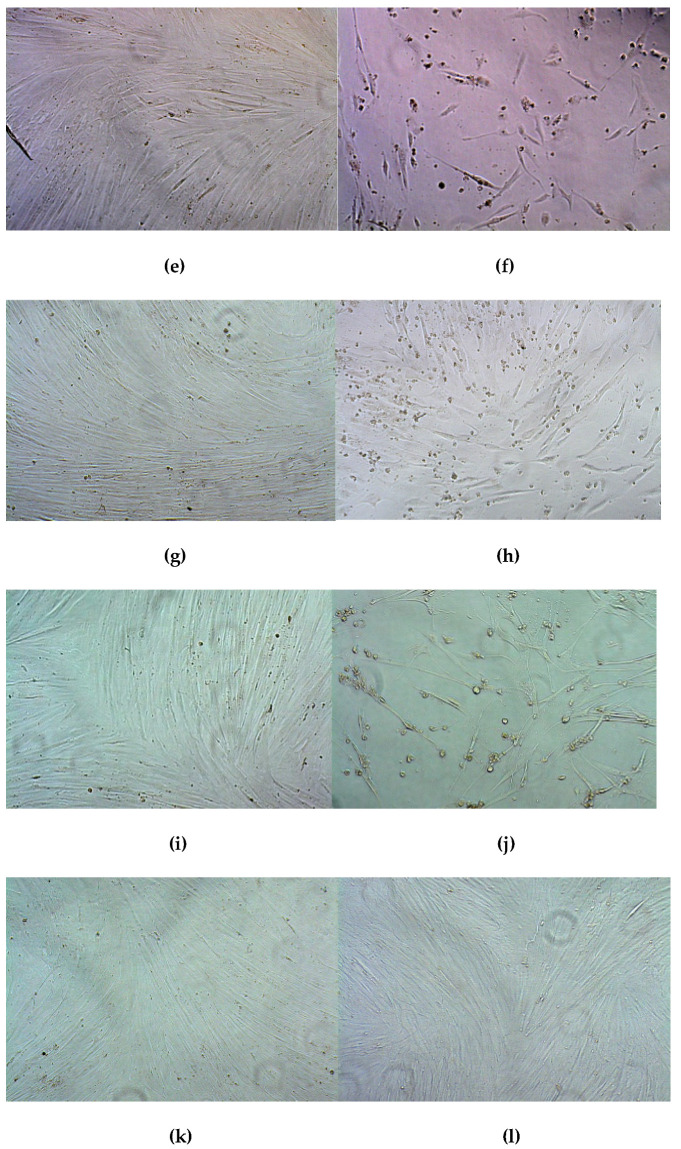
Typical microphotographs in bright field done 96 h after GCV treatment. The UL cells were transfected with RGD1-R6/pCMV-lacZ polyplexes at N/P ratios of (**a**) 8/1, (**c**) 12/1; with RGD1-R6/pPTK1 complexes at (**b**) 8/1, (**d**) 12/1 charge ratio; with RGD1/pCMV-lacZ polyplexes at N/P ratios of (**e**) 8/1, (**g**) 12/1; with RGD1/pPTK1 complexes at (**f**) 8/1, (**h**) 12/1 charge ratio; with PEI/DNA complexes using (**i**) pCMV-lacZ and (**j**)pPTK1 plasmids. Control wells contained (**k**) GCV treated cells and (**l**) untreated intact cells.

The higher decrease in cell viability could be explained by the so-called “bystander effect”, when GCV from transfected cells migrated to non-transfected ones through gap junctions or by endocytosis of apoptotic vesicles [72]. Previously, the study devoted to suicide gene therapy of glioma demonstrated an enhanced bystander effect observed due the overexpression of the gap junction connexin 43 [73]. On the other hand, no significant decrease in cells proliferative activity was observed after their transfection using pCMV-lacZ plasmid which indicates that GCV did not exert any toxic effect on cells non-transfected with pPTK1 plasmid as well as intact cells. The results also confirmed that the decreased cell viability observed in cells transfected with pPTK1 and treated with GCV was predominantly due to the therapeutic approach. The microphotographs of UL cells visualized the results obtained (Figure 10). We could observe a significant decrease in the number of cells after their treatment with RGD1-R6/pPTK complexes as compared to RGD1-R6/pCMV-lacZ, cells treated with plasmid only and intact cells. Similar microphotographs were obtained for PANC-1 cells (Figure S4).

**Figure 11:**
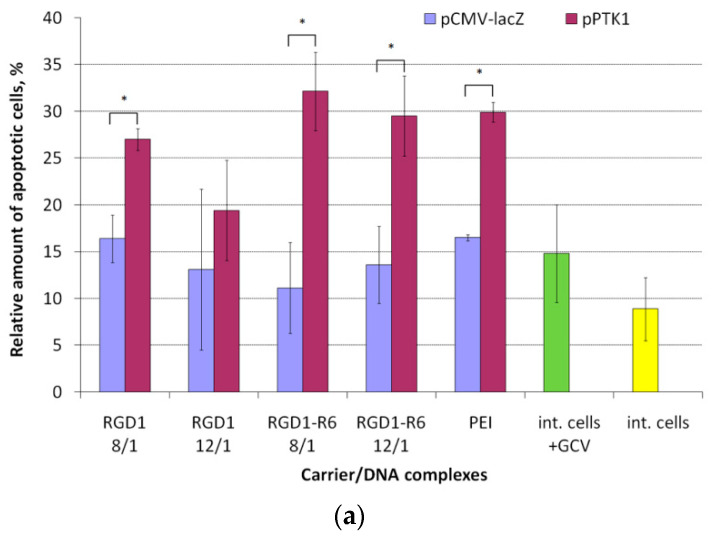

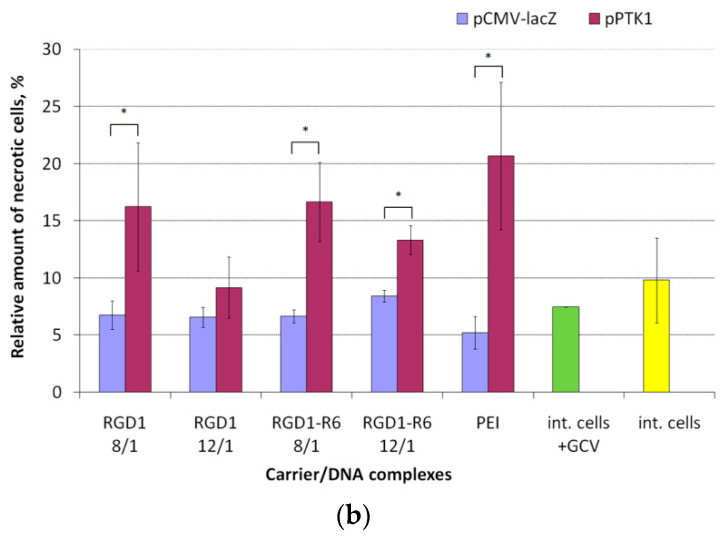
Apoptosis (**a**) and necrosis (**b**) of UL cells induced by GCV treatment after cell transfection with RGD1-R6/DNA or RGD1/DNA polyplexes formed with pPTK and pCMV-lacZ plasmids. Values are the mean ± SEM of the mean of four independent experiments. * *p* < 0.05 compared to pCMV-lacZ-complexes.

By means of annexin V-FITC detection we found that HSV TK expression and GCV treatment triggers apoptosis activation (Figure 11a). It known that apoptosis in the early stages activates processes that include loss of phospholipid asymmetry. In fact, phosphatidylserine normally found on the internal part of the plasma membrane becomes translocated to the external one. Thus, the phosphatidylserine becomes available for the formation of conjugates with annexin V-FITC, which makes possible detection of apoptotic cells [69,74]. The flow cytometry showed that 29–32% of UL cells were annexin V-positive after delivery of pPTK1 plasmid compared to pCMV-lacZ-bearing complexes (11–13%) and intact cells (9–15%) (Figure 11a). The percentage of apoptotic cells transfected with pPTK1 plasmid was 2.1–2.9-fold higher than that of pCMV-lacZ plasmid. Similar results were obtained for PEI/DNA polyplexes (29.9% with pPTK1 versus 16.5% with pCMV-lacZ plasmids). Non-cross-linked RGD1/pPTK1 polyplexes induced only a slight increase in an apoptotic cells number compared to control complexes (1.3–1.7-fold for pPTK1 compared to pCMV-lacZ). The increase in apoptosis in cells transfected by pCMV-lacZ–polyplexes can be explained by the carrier cytotoxicity.

Annexin V detection in PANC-1 cells showed that 26–34% of the cells transfected with RGD1-R6/pPTK polyplexes were positive after 24 h of GCV treatment (Figure S5a). This percentage was significantly differed from that of RGD1-R6/pCMV-lacZ complexes (14.5–18%). Control staurosporine treatment resulted in approximately 50% of annexin V-positive PANC-1 cells (data not shown). PEI-polyplexes demonstrated the same tendency like RGD1-R6/DNA ones, but the percentage of annexin V-positive PANC-1 cells after transfection with PEI/pPTK complexes was slightly lower. On the other hands, non-cross-linking RGD1/DNA polyplexes transfection led to a slight increase in a number of apoptotic cells even for pCMV-lacZ complexes (24–25%). This fact can be explained by the larger amount of ligand in the composition of RGD1 carrier compared to RGD1-R6 carrier, which can cause possible toxic effect on cells associated with cell detaching from substrate via ligand-αvβ3 integrins interaction [65].

In the later stages of apoptosis, the cell membrane becomes damaged, which gives the possibility for intercalating dyes such as ethidium bromide, propidium iodide, etc., to penetrate the cells [74]. We found that the percentage of necrotic cells was lower compared to apoptotic ones (Figure 11b). Only 10–15% of UL cells transfected with RGD1-R6/pPTK or RGD1/pPTK polyplexes were propidium iodide-positive indicating that the cells were mainly in the early apoptosis.

Similarly, 8–11% of PANC-1 cells transfected with RGD1-R6/pPTK or RGD1/pPTK polyplexes were propidium iodide-positive (Figure S5b). Nevertheless, it was significantly higher than percentage of necrotic cells after transfection with pCMV-lacZ complexes. Only for PEI-polyplexes relative number of necrotic cells was higher than apoptotic cells even for PEI/pCMV-lacZ complexes (9% of apoptotic and 13% of necrotic cells). This result demonstrates cumulative decrease in cell viability not only by suicide effects but also by PEI-mediated cytotoxicity [75]. Thus, after 24 hours of GCV incubation, RGD1-R6/DNA polyplexes induced the HSV-TK specific cell death and PANC-1 cells, as well as UL cells, were registered mainly in the early apoptosis rather than necrosis stage.

To sum up, RGD1-R6/pPTK1 polyplexes were found to be safe, highly specific and efficient tools for suicide gene therapy of uterine leiomyoma because of their ability to decrease the proliferation and number of living cells as well as triggering cell apoptosis.

## 4. Conclusions

The current study presents iRGD ligand-conjugated cysteine-rich peptide carrier RGD1-R6 for targeted DNA delivery to αvβ3 integrin-expressing cells. Physico-chemical and cell transfection experiments confirm important role of cysteine modification of the peptide carrier. The DNA/RGD1-R6 complexes exhibited αvβ3 integrin-specific uptake by the cellsdemonstrated by competitive transfections with free cyclic RGD ligand. The thymidine kinase encoding plasmid delivery to cancer and UL cells followed by GCV treatment resulted in significant suicide gene therapy effects. HSV-1 thymidine kinase gene expression in uterine leiomyoma cells reduced their proliferative activity and increased number of apoptotic and necrotic cells. The obtained findings, taken together, allow us toconcludethat the developed RGD1-R6 carrier can be considered as promising tool for suicide gene therapy of uterine leiomyoma.

## Figures and Tables

**Table 1:** Design and composition of the carriers.

Carrier	Composition (mol%)
RGD0-R6	RRRRRRRRRHHHH (50 mol%) + CHRRRRRRHC (50 mol%)
RGD1-R6	RRRRRRRRRHHHH-CRGDRGPDC (50 mol%) + CHRRRRRRHC (50 mol%) |__________|

**Table 2:** Size and zeta-potential of the carrier/DNA complexes.

Carrier	Charge Ratio	Size (nm) ± S.D.	ʐ-Potential (mV) ± S.D.
RGD1-R6	4/1 8/1	304.8 ± 0.69 98.8 ± 0.49	4.7 ± 0.7 16.2 ± 0.1
	12/1 16/1	102.0 ± 0.38 121.2 ± 0.25	24.0 ± 0.3 25.2 ± 0.1
RGD0-R6	4/1 8/1	308.0 ± 0.35 97.5 ± 0.45	5.1 ± 0.6 20.2 ± 0.5
	12/1 16/1	101.1 ± 0.51 129.6 ± 0.42	25.1 ± 0.5 26.9 ± 0.2

## Data Availability

The data presented in this study are available on request from the corresponding author. The data are not publicly available due to restrictions of the subjects’ agreement.

## Supplementary Materials

The following are available online at https://www.mdpi.com/1999-4923/13/2/202/s1, Figure S1: Molecular structure of RGD1, RGD0, and R6 peptides, Figure S2: Transfection efficacy evaluation of RGD1-R6 and RGD0-R6-polyplexes in serum-present conditions, Figure S3: PANC-1 cell viability after HSV thymidine kinase expression and GCV treatment, Figure S4: Microphotographs of PANC-1 cells after GCV treatment, Figure S5: Apoptosis and necrosis of PANC-1 cells induced by GCV treatment after cell transfection.
