# Supplementary material for: Development of iRGD-Modified Peptide Carriers for Suicide Gene Therapy of Uterine Leiomyoma
Source: Pharmaceutics. 2021 Feb 2;13(2):202. doi: 10.3390/pharmaceutics13020202 (PMC7913275; doi:10.3390/pharmaceutics13020202)
Supplement: Supplementary file 1 [file pharmaceutics-13-00202-s001.pdf]

*pharmaceutics*

# Supplementary Materials: Development of iRGD-Modified Peptide Carriers for Suicide Gene Therapy of Uterine Leiomyoma

**Anna Egorova, Sofia Shtykalova, Alexander Selutin, Natalia Shved, Marianna Maretina, Sergei Selkov, Vladislav Baranov and Anton Kiselev**

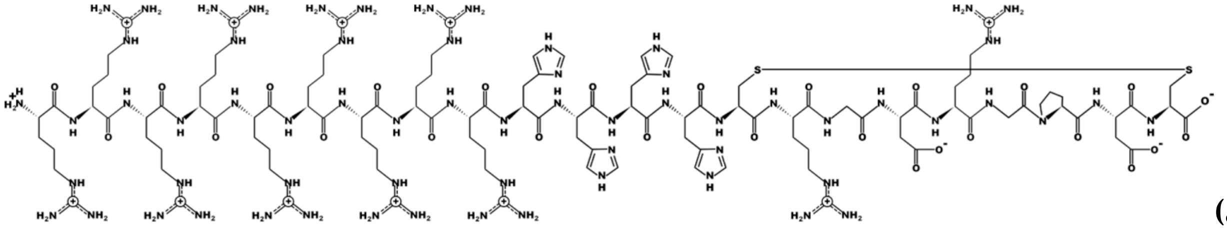

**(a)**

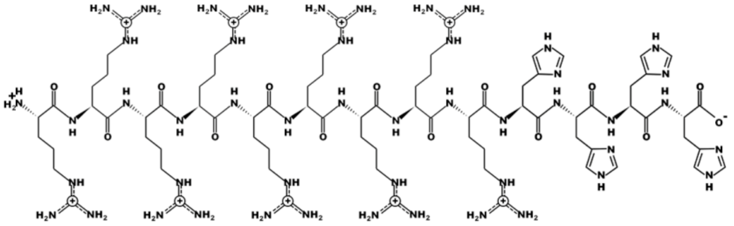

**(b)**

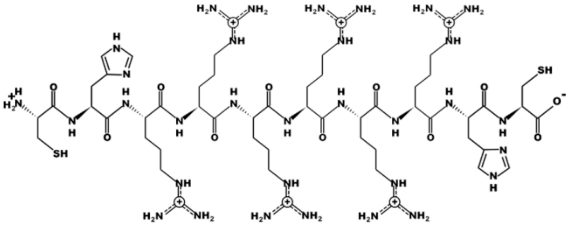

**(c)**

**Figure S1.** Molecular structures of RGD1 **(a)**, RGD0 **(b)**, and R6 **(c)** peptides. The images were processed by PepDraw software (http://pepdraw.com/).

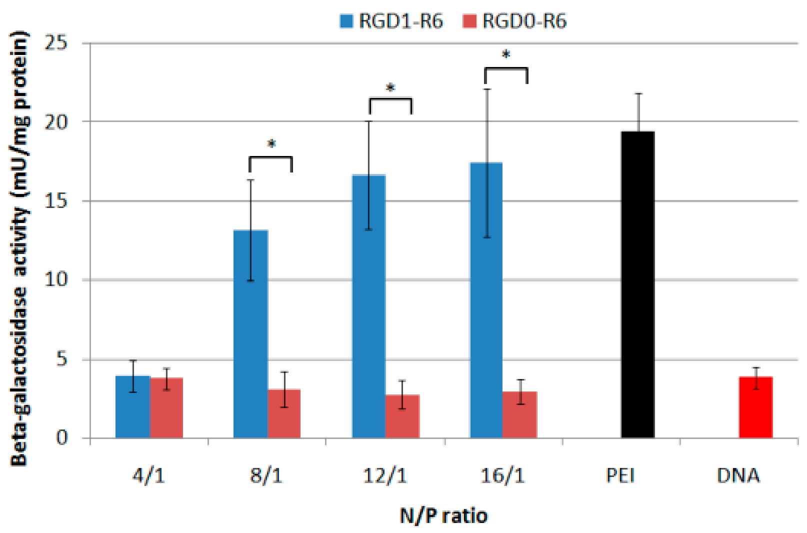


**Figure S2.** Transfection efficacy evaluation of RGD1-R6 and RGD0-R6-polyplexes formed with pCMV-lacZ plasmid in PANC-1 cells in presence of fetal bovine serum. * $p < 0.05$ compared to RGD0-polyplexes.

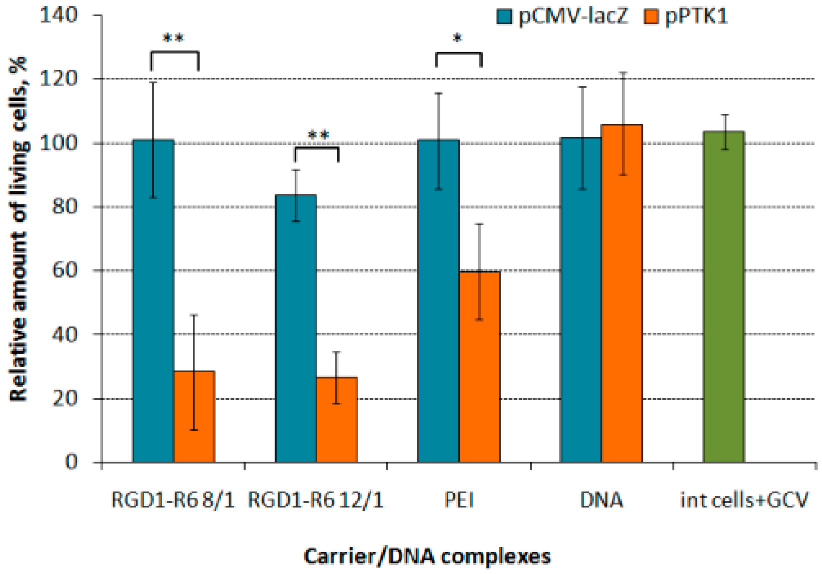


**Figure S3.** PANC-1 cell viability after HSV thymidine kinase expression and GCV treatment. Values are the mean ± SD of the mean of triplicates. * $p < 0.05$, ** $p < 0.01$ compared to pCMV-lacZ-complexes.

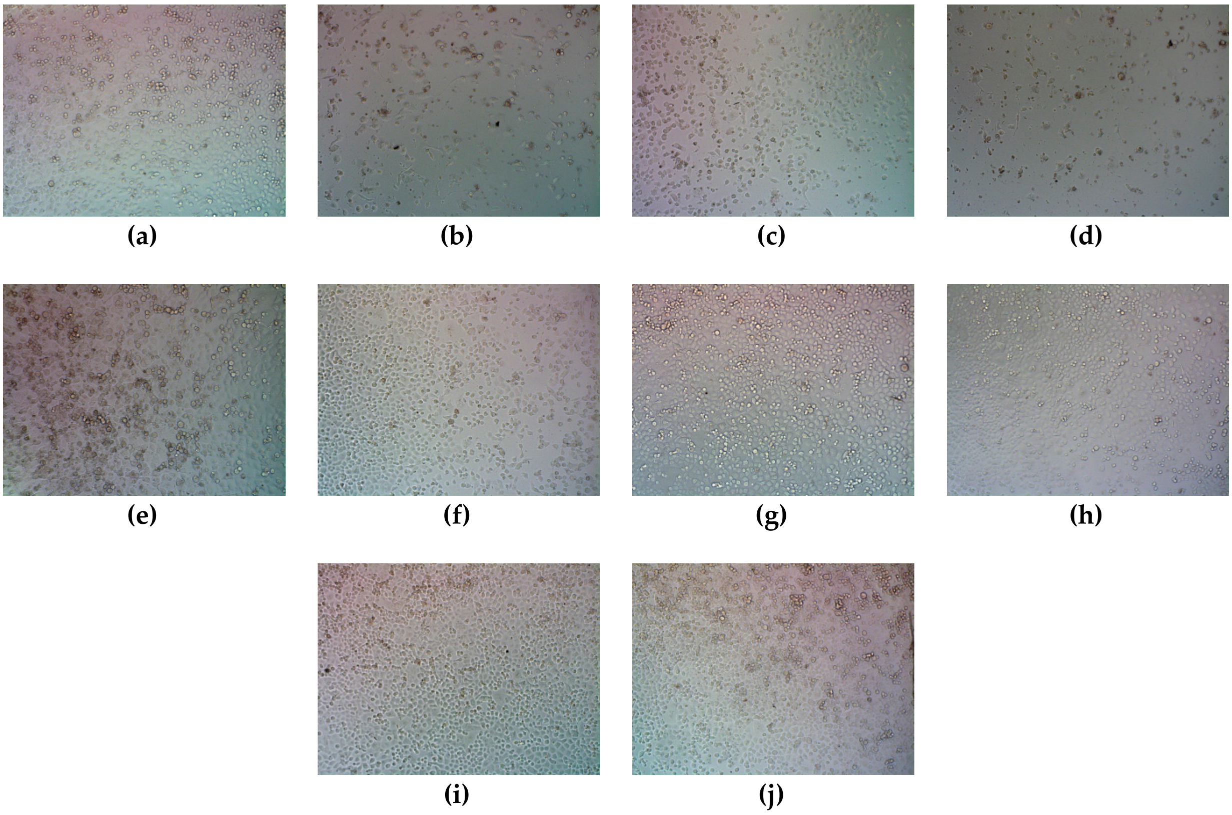


**Figure S4.** Typical microphotographs in bright field after 96 h of GCV treatment. The PANC-1 cells were transfected with RGD1-R6/pCMV-lacZ polyplexes at N/P ratios of **(a)** 8/1, **(c)** 12/1; with RGD1-R6/pPTK1 complexes at **(b)** 8/1, **(d)** 12/1 charge ratio; with PEI/DNA complexes using **(e)** pCMV-lacZ and **(f)** pPTK1 plasmids; with **(g)** pCMV-lacZ and **(h)** pPTK1 plasmids only. Control wells contained **(i)** GCV treated intact cells and **(j)** untreated intact ones.

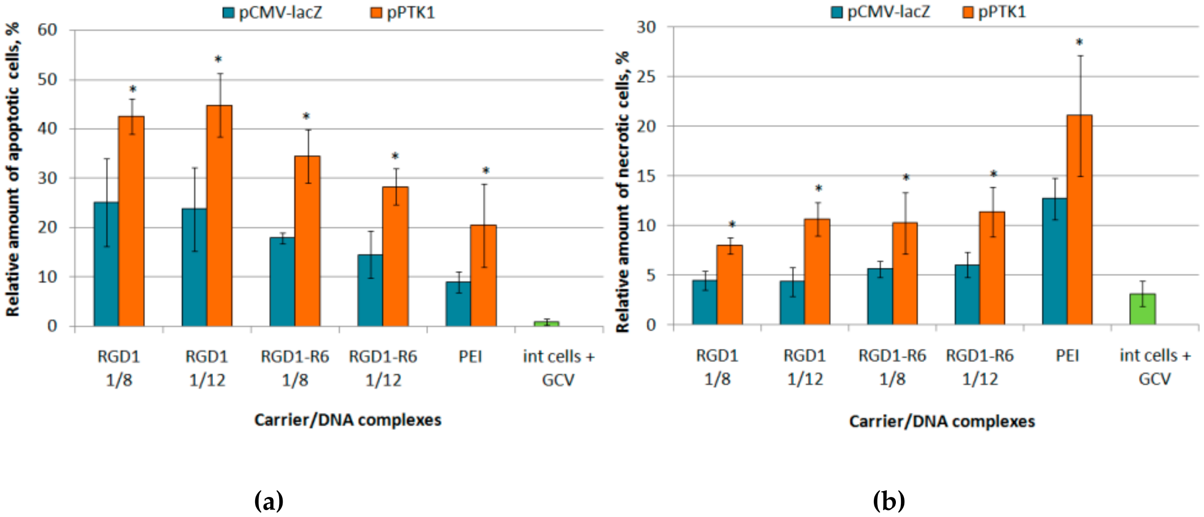


**Figure S5.** Apoptosis **(a)** and necrosis **(b)** of PANC-1 cells induced by GCV treatment after cell transfection with RGD1-R6/DNA or RGD1/DNA polyplexes formed with pPTK and pCMV-lacZ plasmids. Values are the mean ± SEM of the mean of four independent experiments. * $p < 0.05$ compared to pCMV-lacZ-complexes.
